# Crosstalk Between Gut Microbiota and Innate Immunity and Its Implication in Autoimmune Diseases

**DOI:** 10.3389/fimmu.2020.00282

**Published:** 2020-02-21

**Authors:** Yuhao Jiao, Li Wu, Nicholas D. Huntington, Xuan Zhang

**Affiliations:** ^1^The Ministry of Education Key Laboratory, Department of Rheumatology and Clinical Immunology, Peking Union Medical College Hospital, Chinese Academy of Medical Sciences and Peking Union Medical College, Beijing, China; ^2^School of Medicine, Tsinghua University, Beijing, China; ^3^Institute for Immunology, Tsinghua University, Beijing, China; ^4^Tsinghua-Peking Joint Centre for Life Sciences, Beijing, China; ^5^Beijing Key Laboratory for Immunological Research on Chronic Diseases, Beijing, China; ^6^Department of Biochemistry and Molecular Biology, Biomedicine Discovery Institute, Monash University, Clayton, VIC, Australia; ^7^Clinical Immunology Centre, Medical Epigenetics Research Centre, Chinese Academy of Medical Sciences and Peking Union Medical College, Beijing, China

**Keywords:** gut microbiota, innate immunity, innate lymphoid cells, rheumatoid arthritis, spondyloarthritis, systemic lupus erythematosus

## Abstract

The emerging concept of microbiota contributing to local mucosal homeostasis has fueled investigation into its specific role in immunology. Gut microbiota is mostly responsible for maintaining the balance between host defense and immune tolerance. Dysbiosis of gut microbiota has been shown to be related to various alterations of the immune system. This review focuses on the reciprocal relationship between gut microbiota and innate immunity compartment, with emphasis on gut-associated lymphoid tissue, innate lymphoid cells, and phagocytes. From a clinical perspective, the review gives a possible explanation of how the “gut microbiota—innate immunity” axis might contribute to the pathogenesis of autoimmune diseases like rheumatoid arthritis, spondyloarthritis, and systemic lupus erythematosus.

## 1. Microbiota and Human Health

Single-cell organisms were pioneers in the evolution of the earth's ecosystem. Bacteria, as one most crucial domain, have existed for over three billion years (1). The co-evolution of bacteria and other microorganisms with their multi-cell hosts through time gradually built up the unique micro-ecosystem, i.e., microbiota (2). The human body is not a closed system, nor sterile. Microorganisms may implant on open surfaces, such as skin (3, 4), gastrointestinal (5), respiratory (6), and urogenital tract (7), and develop into local microbiota with distinctive features. Overall, microbiota is sharing survival niche with their hosts. They display characteristics like co-adaptation to environment and co-dependency with hosts, and has unique and crucial roles in human physiological and pathological processes (8).

The co-existence of microbiota and human beings has been long. However, numerous medical studies in the past regarded human body as an independent multi-cell organism and neglected the function of microbiota residing in. Lederberg et al. raised the concept of microbiota for the first time and implicated the possibilities that these microorganisms were related to health and disease (9). In the 1960s, with the establishment of germ-free (GF) animal model (10, 11), began the history of unveiling the relationship between gut microbiota and the innate immune system. When exploiting the advantages of GF animal models, people discovered that gut microbiota have critical roles in constructing the structure of gut-associated lymphoid tissues (GALTs) and maintaining their physiological function (12–14). The second landmark was the discovery of pattern recognition receptors (PRRs) superfamily. PRRs mediate the non-specific recognition of exogenous microorganisms by innate immune cells and intestinal epithelial cells (IECs) (15). The discovery enabled us to understand the molecular machinery in the reciprocal interaction between gut microbiota and innate immune system. At present, the most cutting-edge advancements in high-throughput molecular strategies, such as 16S ribosomal RNA sequencing (16–19) and metagenomics (20–22), allow people to study the gut microbiota systematically and genome-widely. An impressive amount of bioinformatic data of skin or mucosal microbiota community organization, in combination with metatranscriptomics and macrometabolomics, strongly promote investigations into the mechanisms of “microbiota—immunology—disease.”

With accumulating studies and deeper understandings, people gradually realized that human individuals are more than multi-cell organisms. The concept of superorganism was then introduced (23): every individual of human beings contains significant numbers of non-human cells, and also microbial genomes that are hundreds or thousands times larger than human genomes (24). How these co-existing microorganisms impact human beings has led to broad and deep discussion among disciplines including microbiology, immunology, oncology, and rheumatology.

Of all the mucosae where microbiota reside, the gastrointestinal tract has the largest mucosal surface. Accordingly, the largest community of microorganisms with the greatest diversity resides in the gut, which is estimated to consist of over 500 different species (25). The total number could reach 10^14^–10^15^, which is ~10–100 times larger than the scale of human body cell number (26). Characterization of the composition of gut microbiota using technologies such as 16S RNA sequencing have shown that the major gut bacterial phyla in healthy human are the *Firmicutes* and *Bacteroidetes* phyla, other species including the *Proteobacteria, Actino-bacteria, Fusobacteria*, and *Verrucomicrobia* phyla are relatively fewer (27). Gut microbiota have unique roles in assisting digestion (28, 29), synthesizing nutrients (30–32), regulating host defense, and facilitating the development of the immune system. At the meantime, environment, diet, and host immune system can impact gut microbiota *vice versa* (2). The reciprocal “host-gut microbiota” axis is critical in keeping local homeostasis and might also contribute to the pathogenesis of certain autoimmune diseases including rheumatoid arthritis, spondyloarthritis, and systemic lupus erythematosus via remodeling the gut immune system (33). The innate immune compartment, serving as the frontline between host and gut microbiota, is tightly related to the gut microorganisms (34). Deciphering the crosstalk between gut microbiota and the innate immune system might shed light on various unknown causes of diseases. This review focuses on the regulatory mechanisms of gut microbiota and the innate immune system, and possible implications to explain the pathophysiology of autoimmune diseases.

## 2. The Role of Gut Microbiota in the Establishment and Homeostasis of Innate Immune System

### 2.1. Gut-Associated Lymphoid Tissues (GALTs) and Mucosal Defense

GALTs are part of the mucosa-associated lymphoid tissues (MALTs) (35), lining directly between host and environment. As the frontline of gut mucosal defense, the principal function of innate immune cells in GALTs is non-specifically recognizing pathogens, initiating innate immune response, and presenting antigens to activate the downstream adaptive immune system. GALTs are also crucial in maintaining the immune tolerance to commensal flora. The dual function of GALTs is critical to the homeostasis between gut microbiota and human immune system.

The histological components of GALTs mainly includes Peyer's patches, crypt patches, isolated lymphoid follicles (ILFs), appendix, and mesenteric lymph nodes (mLNs) (35, 36). Constituent cells of GALTs include M cells, which are capable of transferring antigens but not processing or presenting them (37), conventional lymphocytes such as helper T cells (Th cells) (38, 39), regulatory T cells (Tregs) (40, 41), cytotoxic T lymphocytes (42), IgA producing B cells (43), phagocytes including dendritic cells (40, 41), macrophages (44), and other non-conventional lymphocytes such as innate lymphoid cells (ILCs) (45, 46).

Various studies revealed that the structural build-up of GALTs relies on gut microbiota. The formation of gut secondary lymphoid organs such as Peyer's patches, mLNs and ILFs greatly depends on lymphoid tissue inducer (LTi) cell, a subset of group 3 ILCs, and its crosstalk with the colonization of gut microbiota (47, 48). In GF animals, the development of GALTs is disturbed, typically characterized by aberrant formation of crypt patches and ILFs (49–52). A complete absence of commensal flora in GF mice does not result in an absence of Peyer's patches, yet the sizes of Peyer's patches and germinal centers are significantly reduced in GF mice than those in SPF mice (53). However, reconstitution of gut microenvironment in GF mice via bacteria inoculation could rebuild the structures of GALTs (12). These findings support the conclusion that gut microbiota instructs the structural development of GALTs.

GALTs are the critical link between the local immune response to gut microbiota and systemic immune response. How local immune cells affect distal organs such as joints, bones, skins, pancreatic islets, and even central nervous system remained unspecified. Possible mechanisms could involve the local resident and migrative antigen presenting cells and also circulating adaptive immune cells, which are to be discussed in the following sections. Moreover, there was evidence that different baseline of pro-inflammatory cytokines produced by GALTs from different strains of mice that had different species of gut microbiota might contribute to the susceptibility to autoimmune diseases (54).

The molecular mechanism underlying was elucidated after the discovery of PRRs. The development of ILFs relies on the recognition of pathogen-associated molecular patterns (PAMPs) on the enteric bacteria via certain PRRs and activation of downstream signaling pathways. Various PRR-deficient mouse models have impaired development of ILFs in colon and ileum. So far, multiple PRR-related molecules are found to be involved in the mechanism include toll-like receptors 2 (TLR2) (55), nucleotide-binding oligomerization domain 1/2 (NOD 1/2) (49, 56, 57), myeloid differentiation primary response 88 protein (MyD88) (58, 59), and TIR domain-containing adaptor protein inducing interferon (IFN)-β (TRIF) (49).

**Figure 1:**
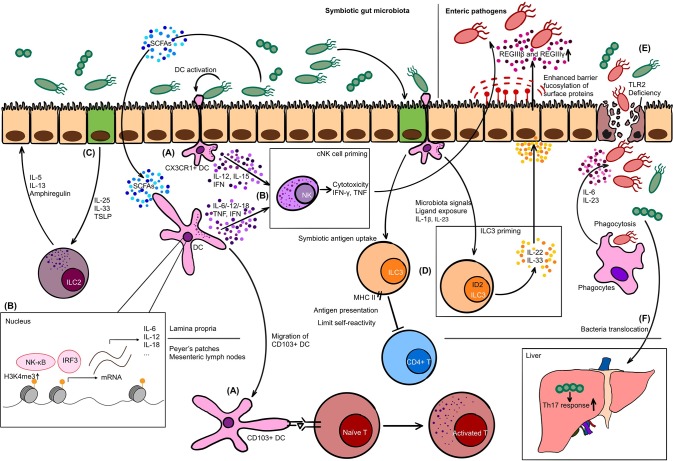
The interplay between innate immune cells and gut microbiota. **(A)** At steady state, CX_3_CR1+ dendritic cell (DC) forms dendrites for phagocytosis, while CD103+ DC migrates to Peyer's patches or mesenteric lymph nodes to present antigens to naïve T cells. **(B)** Upon activation by commensals, DC secretes interleukin (IL)-12, IL-15, and interferon (IFN) to prime conventional NK (cNK) cells. Short-chain fatty acids (SCFAs) as metabolic by-products upregulate H3K4me3 in DC and promote the production of IL-6, IL-12, IFN, and tumor necrosis factor (TNF), which is another strategy to condition cNK cells. Conditioned cNK cells have proper cytotoxicity and cytokine secretion capability to exert the anti-microbial or anti-viral function. **(C)** Intestinal epithelial cell (IEC) in response to commensal bacteria produces IL-25, IL-33, and thymic stromal lymphopoietin (TSLP) to activate ILC2. **(D)** Major histocompatibility complex class II (MHC II) expressing ILC3 is capable of presenting commensal antigens to CD4+ T cells, limiting their self-reactivity. The priming of ILC3 also relies on microbiota signals in an ID2-dependent manner. Primed ILC3 secretes IL-22 and joins the defense against pathogens: driving the antimicrobial peptide production such as REGIIIβ and REGIIIγ, and fucosylation of surface proteins on IEC. **(E)** TLR2 is crucial in maintaining the integrity of IEC. Deficiency may increase the susceptibility of intestinal inflammation. Invasive pathogens, in this case, invade into lamina propria layer. Phagocytes exert host defense function via phagocytosis and cytokine production such as IL-6 and IL-23. **(F)** Translocation of commensal bacteria *Enterococcus gallinarum* from gut to liver enhance the Th17 response and worsen manifestations of autoimmune diseases.

PRR-PAMP recognition of commensals not only drives the development of the structure of GALTs, but also plays a substantial role in conditioning the host defense function. Gut commensal flora prime Peyer's patches via TLR pathways to promote the production of antimicrobial peptides like REGIIIβ and REGIIIγ. In turn, inhibition of TLR pathway-related molecules could increase the susceptibility of enteric pathogen infection (60, 61). More specifically, the deficiency of TLR2 impairs the integrity of intestinal epithelial barrier, which breaks the balance between commensals and host defense and aggravates colitis (62) (Figure 1E). Besides, compiling studies showed that metabolic by-products produced by symbiotic bacteria such as short-chain fatty acids (SCFAs) regulate the immune reaction of GALTs via epigenetic mechanisms to exert their roles of host defense and maintenance of immune tolerance to commensals through. The details are to be discussed in the following chapters.

In conclusion, gut microbiota shapes the structural development of GALTs and primes its immune response to initiate the host defense function and to maintain tolerance against commensal bacteria, via PRR-PAMP recognition and epigenetic modulators like SCFAs. In the pathogenesis of autoimmune diseases, GALTs, especially mesenteric lymph nodes were the initial sites to induce the gut-driven immune responses (63), and possibly lead to the change of immune response systemically (64).

### 2.2. Innate Lymphoid Cells

Innate lymphoid cells (ILCs) are essential constituent members of the innate compartment of GALTs. The most significant feature that distinguishes them from T or B cells is that ILCs do not require antigen receptor gene rearrangements mediated by recombinant activating gene (RAG) for development (65). Hence, ILCs do not express antigen-specific receptors such as T cell receptors or B cell receptors, but possess a spectrum of surface receptors including activating, inhibitory, and cytokine receptors that senses the “pattern” of the immune niche, to further determine their fate—whether to become killers or become silent (66).

It is intriguing that the subpopulations of ILCs share certain immunological characteristics with helper T (Th) cells (67). The current nomenclature for ILCs are based on their determinant transcription factors and signature cytokines. Hence, ILCs are categorized into three major subpopulations. Group 1 ILCs, like Th1 cells, are T-bet (i.e., TBX21)-dependent, secreting the signature cytokine interferon (IFN)- γ (68–70). Group 2 ILCs, like Th2 cells, are categorized by their GATA3-dependency and the ability to secrete interleukin (IL)-5 and IL-13 (71–73). Group 3 ILCs, like Th17 and Th22, are dependent on the transcription factor RORγt and secrete signature cytokines IL-17 and IL-22 (74, 75). Group 3 ILCs consist of the CD4+ CD3– CCR6+ subset, namely LTi cells, and the ILC3 subpopulation that do not express the tissue homing factor CCR6 (74). Moreover, recent studies in these 10 years have revealed the heterogeneity of group 1 ILCs, which consist of the cytotoxic and the non-cytotoxic compartment, which are conventional natural killer (cNK) cells and ILC1, respectively (76).

Instead of circulating in the periphery, majority of ILCs reside in non-lymphoid tissues, especially epithelial tissues such as skin, gastrointestinal tract, respiratory tract, salivary glands, and non-epithelial organs such as liver (77). In different tissues, ILCs display heterogeneity on both transcriptome and immune function level (78), consolidated by abundant functional analyses and single-cell RNA sequencing (79, 80). The puzzle why ILCs have such heterogeneity has long been mysterious. It was proposed that exposure to different local microbiota or external intakes might contribute to shape the heterogeneity of local ILC populations (81, 82).

#### 2.2.1. Conventional Natural Killer Cells

Conventional natural killer (cNK) cells are recognized as the only cytotoxic population of ILCs. They are unique for their capability to distinguish “non-self” from “self” via the signaling pathway system comprised of activating receptors and inhibitory receptors (83).

The cNK cell pool consists of a circulating compartment and the other tissue-resident compartment. A significant proportion of cNK cells circulate in the blood and primary lymphoid organs like bone marrow and spleen (84). As to the tissue-resident cNK cells in the gut intraepithelial layer and lamina propria layer, they share the most of biological features with other cNK cell compartments. cNK cells are able to sense pathogens, oncogenesis, and tissue damage signals. Activation and turnover of cNK cells rely on the overall signal input of activating signals, inhibitory signals, and exogenous cytokine signals, which further leads to the alteration of specific transcription factors and a group of pro-apoptotic proteins and ultimately determines the fate of cNK cells (85). Upon activation, cNK cells exert the cytotoxicity function via releasing the pore forming cytolytic protein–perforin and the cytotoxic protein–granzyme. Besides, tumor necrosis factor (TNF)-related apoptosis-inducing ligand (TRAIL) pathways and antibody-dependent cellular cytotoxicity (ADCC) are also cytotoxic strategies cNK cells adopted (86). At the same time, cNK cells possess strong cytokine production ability, including TNF, IFN-γ, and granulocyte-macrophage colony-stimulating factor (GM-CSF) (87).

However, the mechanism that cNK cells employ to maintain the immune balance between allogeneic reactivity and self-tolerance has long been mysterious. The “missing-self” theory that cNK cells recognize exogenous cells by detecting decreased expression of major histocompatibility complex (MHC) class I could only partially explain the mechanism. Another theory of cNK cell “education” was then proposed to clarify the activation and immune regulation mechanisms. Through the development of cNK cells, the threshold of activation is modulated by adjusting the expression level of activating receptors and inhibitory receptors, which is termed as “education.” Another term “arming” describes the downregulation of inhibitory receptors that could upregulate the threshold of activation. On the contrary, the term “licensing” depicts the scenario where activating receptors are downregulated to endow cNK cells with increased receptivity to activating signals. The arming and licensing process of cNK cells is crucial to ensure the appropriate activation strategy, namely, to limit self-reaction of cNK cells that do not recognize self MHC class I molecules via inhibitory receptors. Generally, educated cNK cells, marked by the elevated expression of the activating receptor DNAM-1, exhibit higher reactivity to missing-self targets with increased degranulation and cytokine production capability (88). Related research of cNK cell education theory was mainly in the settings of virus infection and oncoimmunology. There was a lack of elaborative studies of cNK cell licensing or arming in autoimmune disease models. However, aberrant phenotypes of cNK cells in various autoimmune diseases were already reported. cNK cells manifested impaired cytotoxicity and decreased cNK cell frequency in the periphery in autoimmune diseases like systemic lupus erythematosus (SLE), rheumatoid arthritis (RA), and multiple sclerosis (89, 90). At the meantime, cNK cells accumulate in inflamed sites, such as synovial fluid in RA patients (91). These cNK cells, however, present stronger cytotoxicity and cytokine production capability comparing to the circulating cNK cells. The significance of the differential phenotype of cNK cells between the periphery and affected tissue remains uncertain with respect to the pathogenesis of autoimmune diseases. Whether these cNK cells in autoimmune diseases lose their tolerance to “self” tissue is also unspecified. The findings of killer immunoglobulin-like receptors (KIRs), a family of activating receptors of cNK cells, might give a clue. Polymorphism of KIR genes in various autoimmune diseases has been reported. The high expression of KIRs together with downregulation of cNK cell inhibition (an unlicensed phenotype) were related to incidence of autoimmune diseases, which indicated that altered cNK cell education might be related to the pathogenesis.

Acquisition of an educated phenotype of cNK cells in diseases is still under exploration (92). However, gut microbiota contains a large pool of ligands to cNK cell receptors, which might be one of the centers for cNK cells to obtain normal function and acquire education. Lack of over-exposure to pathogens, cNK cells from GF mice have limited cytotoxicity and cytokine production (93). Gain of cytotoxic function of cNK cells is therefore found to be dependent on the priming step by commensal bacteria in a dendritic cell dependent manner (94). And the commensal bacteria lactic acid bacteria (LAB) were also found to be a key regulator in the cross talk between cNK cells and dendritic cells. LAB activates immature dendritic cells in the gut to produce key cytokines including IL-12 and IL-15, and to favor the activation and proliferation of cNK cells (95) (Figure 1B).

Collectively, current knowledge indicates that commensal bacteria were critical in priming cNK cells. The education theory lacks specific elaboration in this setting but might be crucial in the pathophysiology in autoimmune diseases. However, future studies are required to determine the specific role of cNK cells, especially the function of activating and inhibitory receptors that can be affected by gut microbiota, in the pathogenesis of autoimmune diseases.

#### 2.2.2. Helper-Like ILCs

Other ILC populations except cNK cells possess no cytotoxic function. Due to their transcriptional and cytokine characteristics corresponding to helper T cells, ILC1, ILC2, ILC3, and LTi cells are therefore categorized as the helper-like ILCs. Gut helper-like ILCs are the primary compartment of ILCs in the gut. The development of these ILCs has shown to be independent on gut commensal flora according to the studies of GF and SPF mice (96). However, the immunological function such as cytotoxicity and cytokine production might require the existence of gut microbiota (97, 98). Majority of the research into gut microbiota and ILCs focused on ILC3 owing to its high frequency and broad distribution at the interface of microbiota-gut. ILC3 is a critical RORγt-dependent ILC subset in producing the cytokine IL-22, which has pivotal roles in maintaining the survival and proliferation of epithelial cells and the production of antimicrobial peptides (99).

The differentiation of ILC3 and production of IL-22 were initially found to rely on the existence of symbiotic enteric bacteria (97). On the contrary, an opposing study claimed that the symbiotic bacteria could inhibit ILC3 from producing IL-22 but not affect the differentiation of ILC3 (100). Meanwhile, other studies emphasized on the reciprocal interaction between ILC3 and enteric bacteria. Metabolic by-products from lactobacillus could activate aryl hydrocarbon receptor (AHR) and prime the IL-22 capability of ILC3 (101). Reciprocally, IL-22 produced by ILC3 was firstly shown to be the major the mechanism of host defense against pathogenic bacteria *Citrobacter rodentium* (98, 102). Recently, the process was further found to be regulated in an ID2 dependent manner. ID2, together with RORγt, is the key transcription factor determining the fate of ILC3. Moreover, ID2 is essential for ILC3 to produce an adequate amount of IL-22 via the IL-23R pathway by commensal bacteria. Deficiency of ID2 in ILC3 significantly reduces the production of IL-22 and fails to maintain the homeostasis of gut microbiota (103) (Figure 1D). In the *Il22*-deficient or IL-22-secreting-ILC-deficient models, the production of antimicrobial peptides like REGIIIβ and REGIIIγ is downregulated (104), and the overgrowth, bacterial transposition, and dissemination of a potential pathogenic species *Alcanligenes xylosoxidans* consequently happen (105) (Figure 1D). These studies indicate that commensal flora is crucial in induction of the host defense function of ILC3, and in turn, to protect the host from pathogens.

Besides affecting the antimicrobial role of ILC3, the gut commensal flora is also involved in the interaction between ILC3 and other immune compartments or even non-immune cells, leading to a vast downstream effect (106), including both host defense and immune tolerance. The soluble lymphotoxin (LT)-α3 and membrane-bound LT-β produced by RORγt+ ILC3 induces the secretion of mucosal associated IgA, which contributes to host defense and maintenance of the homeostasis of commensal flora (107). Besides, IL-22 produced by ILC3 triggered by commensals could stimulate the expression of fucosyltransferase 2 in the IECs and further upregulates fucosylation level of the proteins on luminal surface. This mechanism is critical to enhance the physical host defense barrier against pathogenic microorganisms and strengthen the integrity of gut mucosa (108). Another study has demonstrated the role of commensals on promoting the differentiation of a RORγt^+^ NKp46^+^ NK1.1^hi^ unconventional NK cells that produces IL-22 but not IL-17. This might indicate that dysbiosis in the gut could potentially interfere with the IL-22 and IL-17 production (97). In regard to tolerance induction, ILC3 are able to present antigens from symbiotic bacteria to CD4+ T cells, which in turn can limit the adaptive immune response against symbiotic bacteria. The interaction between ILC3 and CD4+ T cells gives another possible mechanism that explains the sophisticated immune tolerance between flora and immune response (109, 110).

All the studies described above suggest that gut commensal flora prime the ILC3 to refine their normal function such as cytokine secretion and antigen presentation. Absence or aberrance of gut microbiota could lead to the disturbance of ILC3 function and further cause pathogen over-proliferation or breakdown of immune tolerance, which again could possibly contribute to the pathogenesis of autoimmune disease, yet to be consolidated by future research. Meanwhile, how gut flora regulates the function or development of ILC3 still lack a particular explanation. Also, the symbiotic species of gut flora of human beings are significantly different from those in mice, hence, whether the findings are translatable remained uncertain as well.

LTi cells are another RORγt+ group 3 ILC subpopulation that unique to their expression of CCR6. In the hematopoiesis of all ILCs, LTi cells originate from common helper-like innate lymphoid cell progenitors (CHILP) and diverge early from the developmental tree, marked by downregulation of the transcription factor promyelocytic leukemia zinc finger (PLZF) (111). LTi cells are enriched in fetal lymphoid organs and intestine to induce the formation of secondary lymphoid tissues (47), in a microbiota dependent manner (introduced in the previous chapter). Peptidoglycan from enteric Gram-negative commensals primes LTi cells accumulated in lamina propria layer between intestinal crypts (49). Subsequently, stromal cells in the primitive ILF are activated by primed LTi cells via recognition of LT-β and also directly by commensal bacteria via NOD1 signaling pathway. Activated stromal cells secrete MadCAM-1 and CCL19 to recruit B cells and dendritic cells, respectively, and therefore induce ILF formation (49, 112). On the other hand, microbiota promote the production of CCL20 and β-defensin 3, both of which could in turn activate LTi cells by binding to CCR6 (49). Hence, gut microbiota in the construction of ILFs possibly work as the initializer of a positive feedback loop for the continuous activation of LTi cells and stromal cells to trigger the recruitment of B cells and dendritic cells and ILF development.

As to other helper ILC subsets, ILC1 have extremely low frequency in fetal intestine when gut microbiota have not yet established (68, 113), indicating that the development of ILC1 or homing of ILC1 precursors is dependent on commensal bacteria. Intestinal ILC2 are activated by IL-25, IL-33, and thymic stromal lymphopoietin (TSLP) which are cytokines produced by intestinal epithelium in response to commensal bacteria-derived signals (114) (Figure 1C). Another recent study demonstrated that butyrate, a member of SCFAs, could suppress the production of IL-5 and IL-13 by ILC2 through HDAC inhibition (115).

With the emergence of bioinformatic strategies, comprehensive analyses of the atlas of both microbiota and immune cells become possible. In 2016, a group used the single-cell RNA sequencing, indexing-first chromatin immunoprecipitation (iChIP) and assay for transposase-accessible chromatin (ATAC-seq) technologies to analyze the relationship between microbiota and ILCs in the lamina propria layer of the small intestine. Heterogeneity within ILC populations determined simply by surface markers is found. At the meantime, some unique features of ILC3 identified by previous studies are again confirmed, e.g., the antigen presentation capability of MHC class II-expressing ILC3 that limits T cells recognizing commensal bacteria is identified as the unique feature of an independent subpopulation of ILC3 (110), namely ILC3e according to the nomenclature in this study (Figure 1D). Furthermore, the study proved that the gut microbiota has great impacts on the function and heterogeneity of gut ILCs from both transcriptional and epigenetic levels (116). Depletion of commensal bacteria by either broad-spectrum antibiotics or using GF animals is related to a shift in transcription elements of pan-ILCs toward ILC3 characteristics, which, more specifically, points out the possibility that commensal flora might work as a natural suppressor to ILC3-like immune responses in the gut. This idea might give the insight to explain the pathogenesis of some autoimmune diseases involving Th17/ILC3 related immune disorders.

Overall, maturation of ILCs and their cytokine production are dependent on the exposure to symbiotic bacteria. As to ILC3, its dual function in both host defense against pathogens and induction of immune tolerance of the adaptive immune system is closely related to commensal flora as well. Multiple studies have revealed the impact of gut microbiota on ILC3 and its possible effect on changing the downstream immune response, especially the Th17-associated immune responses. Hence, the gut microbiota-ILCs axis is anticipated to play a crucial role in maintaining the homeostasis of both immune and microbiological system in the gut.

### 2.3. Phagocytes: Macrophages and Dendritic Cells

Another immune cell population closely related to gut microbiota are phagocytes including macrophages, dendritic cells, and other non-immune cells like IECs that are also capable of phagocytosis and antigen presentation. Gut phagocytes have pivotal roles in the maintenance of gut homeostasis, especially in the immune tolerance to symbiotic bacteria and immune recognition of pathogenic bacteria.

Macrophages in the gastrointestinal tract are featured with their tissue-residency. Gut macrophages are mainly distributed within the epithelium layer, intraepithelial layer, lamina propria, and structures of GALTs such as Peyer's patches, isolated lymphoid follicles, and mLNs. Unlike tissue-resident macrophages in other organs such as skin or liver, a large proportion of gut macrophages (Tim-4- macrophages) are directly derived from circulating monocytes. However, recently, the Tim-4+ CD4+ gut macrophages are found to be locally maintained, whereas the Tim-4- macrophages are the subset that joins the peripheral monocyte replenishment cycle (117). The homing and development of these macrophages from the periphery to the gut is directed by the chemokine receptor CCR2, the expression of which is induced by commensal bacteria. The maintenance of a normal gut macrophage population relies on the continuous presence of commensal bacteria. Lack of gut microbiota would cause insufficient chemotaxis signal for local macrophage replenishment (118).

Gut microbiota could also promote the development of peripheral myelocytes, including macrophages, by affecting primitive hematopoiesis of myeloid cells in both yolk sac at early stages and in bone marrow afterwards. An absence of gut commensal flora significantly increases the susceptibility to bacterial infection due to impaired host defense immune response mediated by the myeloid cells (119). Another study indicated that myelopoiesis in the bone marrow is positively correlated to the diversity of gut microbiota and the serum TLR level (120). It was then suggested that the local flora could affect the hematopoiesis process spatial apart via PRR-PAMP pathways.

Dendritic cells share part of the features of macrophages, like their distribution in the gut and function of phagocytosis. On the other hand, dendritic cells are unique to their strong capability of antigen processing and presentation to the adaptive immune system. Dendritic cells in the gut can be categorized into two subsets by the differential expression of CD103 (αE integrin), a chemokine receptor CX_3_CR1, and CD11b (121). With the existence of gut commensal flora, the CD103+ dendritic cells are capable of migrating from the intestine to mLNs (122) and triggering the migration of T cells to gut lumen to induce immune response via chemotaxis process depending on CCR7 (123). Another distinct population, CD103– CD11b+ CX_3_CR1+ dendritic cells, resembling macrophages, has less capability of migration and less efficiency in activating T cells. However, this dendritic cell population is considered to be able to form trans-epithelial dendrites and phagocytose invasive enteric pathogens, and further to process and present antigens (122) (Figure 1A). Conversely, in the case of dysbiosis of gut microbiota, such as in the salmonella infection model, it is CD103+ dendritic cells that are found to accumulate in the enteric epithelium layer and form the trans-epithelial dendrites to phagocytose pathogenic bacteria (124). On the other hand, studies with the dextran sulfate sodium (DSS)-induced colitis model, antibiotics induced colitis model and *Myd88*-knockout mouse model all reach identical conclusions that CX_3_CR1+ dendritic cells have strong ability of migration to mLNs and antigen presentation (120, 125). What can be inferred from above is that in the steady state, commensal flora in the gut “blocks” the migration of CX_3_CR1+ dendritic cells to mLNs to present both commensal and pathogenic antigens (126), however, dysbiosis might disturb the balance and self or commensal flora-associated antigens could be inappropriately presented and would further cause diseases.

From the epigenetic level, other studies find out that the metabolic by-products from gut microbiota like SCFAs could impact the local homeostasis of phagocytes like macrophages and also myelopoiesis in the bone marrow. The principal mechanism of the epigenetic modulation by SCFAs are summarized as direct inhibition of histone deacetylases (HDACs), and activation of G-protein coupled receptors (GPCRs) (127). In GF mice, phagocytes like dendritic cells are not able to induce the production of various pro-inflammatory cytokines including type 1 IFNs, IL-6, IL-12, IL-18, and TNF. The significant alteration observed is on the epigenetic level, instead of changes of conventional signaling pathways and transcriptomes. The study discovered that upon stimulation by pathogens, the PRR signaling pathway and the NF-κB and IRF3 transition to nucleus stay intact in dendritic cells, whereas the degree of trimethylated H3K4 (H3K4me3) is strongly downregulated in dendritic cells from GF mice, which further leads to a failure of NF-κB and IRF3 binding to the promoter of downstream cytokines. In lack of these cytokines, the activation and maturation of T cells and NK cells are also dampened, which might further increase the susceptibility to infection (94) (Figure 1B). As to macrophages, microbiota induces the expression of HDAC3 and upregulates the anti-inflammatory cytokine IL-10 production, due to the increased deacetylation of *Il10* promoters by HDAC3. The study suggests that gut microbiota is crucial in maintaining the balance of pro- and anti-inflammatory cytokines and local inflammation reaction (128). Another study revealed that SCFAs affect the histone acetylation level in gut macrophages in an HDAC-dependent manner, and further cause a downregulation of pro-inflammatory cytokine profile, such as IL-6 and IL-12 (129). The polarization of macrophages is one of the critical regulators of local inflammation response and is also found to be regulated by microbiota-derived SCFAs. Both *in vivo* and *in vitro* studies demonstrated that SCFAs favor the polarization to alternatively activated macrophages, namely M2 macrophages, which mainly exert anti-inflammation function (130). Besides the modulation of epigenetics in local immune cells, SCFAs are strikingly found to be able to shape the hematopoiesis in bone marrow. Enhanced hematopoiesis of phagocytes including macrophages and dendritic cells is observed in mice administered with SCFAs (131).

Another substantial role of antigen presenting cells are their potential of initiating different axis of adaptive immune response after exposure to different species of bacteria. DCs from MLNs of GF mice, compared to SPF mice, had deficient capability in initializing the IL-17 and IFN-γ by T cells, which was believed to explain the protection from autoimmune encephalitis in GF animal models (132). There are studies found that under the activation of different commensal or pathogenic bacterial strains, dendritic cells, or macrophages showed different patterns of activation and triggers different downstream immune responses. A recent mouse model study revealed that GF mice accepting fecal transplantation from lupus mice developed autoantibody and also local inflammation (133). An altered gut immune response was also observed in these recipients comparing to GF mice accepting fecal transplantation from WT donors. It is possible that different species of bacterium could lead to a different presentation of antigens and initiate the autoimmune pathogenesis.

Overall, SCFAs as by-products from gut microbiota serve as an essential modulator in the epigenetics of both local and distant immune system. Current knowledge suggests that these gut microbiota-derived signals are crucial in maintaining the homeostasis of phagocytes and limiting inflammatory responses via various mechanisms. Lack of SCFAs in GF models could result in a higher risk of infection or inflammation-related diseases. Antigen presenting cells could serve as the link between local immunity to microbiota and systemic autoimmunity. Antigens from altered microbiota that mimic self-tissue presented by antigen presenting cells is one critical mechanism, which has already been strongly indicated by metagenomics analysis (134, 135). Moreover, gut microbiota might possibly trigger different local immune response of antigen presenting cells and eventually lead to an altered adaptive immunity.

## 3. The “Gut Microbiota—Innate Immune System” Axis in the Pathogenesis of Autoimmune Diseases

Gut commensal bacteria have vital roles in the establishment of a regular innate immune system. In turn, dysbiosis of gut microbiota might cause the alteration of the innate immune system, and *vice versa*. The reciprocal interaction between gut microbiota and innate immune system has great significance to maintain homeostasis, whereas the aberrance of the interaction might contribute to the particular pathogenic process of diseases (summarized in Figure 1 and Table 1). Autoimmune diseases such as rheumatoid arthritis, spondyloarthritis, systemic lupus erythematosus, etc. are believed to be caused by the malfunctioning immune system, however, with no solid pathogenic mechanism discovered till present. The missing link might be related to microbiota, as there were already studies indicating so. The chapter is to discuss the possibility of how gut microbiota changes the overall immunity and contributes to the pathogenesis of autoimmune diseases via the innate immune system.

**Table 1:** A summary of the main function of different innate immune subsets and their phenotypes related to microbiota alterations.

**Cell type**	**Subset**	**Main function**	**Phenotype related to microbiota alterations**	**References**
ILC	cNK cells	Anti-virus Anti-tumor Cytotoxicity: perforin, granzyme, TRAIL, ADCC Cytokine: TNF, IFN-γ, and GM-CSF	Decreased cytotoxicity and cytokine production in GF mice Microbiota primes cNK cells via dendritic cells epigenetically	(93) (94)
	ILC1	Anti-virus Anti-tumor Cytokine: TNF and IFN-γ	Low frequency of ILC1 in fetal gut Increased ILC3-related elements upon microbiota depletion	(68, 113) (116)
	ILC2	Anti-parasite Wound healing Cytokine: IL-13 and IL-5	Activated by IL-25, IL-33, and TSLP produced by intestinal epithelium in response to commensals Butyrate suppress IL-5/IL-13 production by ILC2 via HDAC inhibition	(114) (115)
	ILC3	Intestinal barrier Anti-virus Anti-bacteria Cytokine: IL-17 and IL-22	Differentiation and IL-22 production are microbiota-dependent Microbiota inhibit IL-22 production by ILC3 ID2-deficiency downregulate IL-22 production of ILC3, resulting in dysbiosis ILC3 process antigens from symbiotic bacteria and restrain self-reactive T cells Microbiota induce IL-22 production of ILC3 to enhance intestinal barrier	(97) (100) (103) (109, 110) (108)
	LTi cells	Induction of secondary lymphoid tissue formation Cytokine: IL-17 and IL-22	Peptidoglycan from Gram-negative bacteria activate LTi enriched in cryptopatches	(49)
Phagocytes	Macrophages	Phagocytosis Antigen presentation M1 macrophage: pro-inflammatory, anti-bacteria M2 macrophage: wound healing, tissue repair, IL-10 production	Microbiota induce CCR2 expression for macrophage homing Microbiota promote myelopoiesis Microbiota drive the expression of HDAC3 to promote IL-10 production SCFAs downregulate pro-inflammatory cytokine in a HDAC-dependent manner	(118) (119) (128) (129)
	Dendritic cells	Antigen presentation Phagocytosis	Dysbiosis cause CD103+ DC accumulation in epithelium In colitis CX_3_CR1+ DC migrate to mLNs to present antigens Microbiota induce trimethylation of H3K4 to induce IL-6 and IFNβ1 secretion	(124) (120, 125) (94)

### 3.1. Rheumatoid Arthritis

Rheumatoid arthritis (RA) is a systemic autoimmune disorder that mainly affects joints. The influential pathological manifestation is synovitis (136). Immunological analyses found various alterations of the phenotype and function of T cells, such as the imbalance between Tregs and Th17 in synovium, synovial fluid and peripheral blood, resulting an aberrant Th17 response and increased IL-17 production (137). Meanwhile, the overproduction of TNF-α and IL-1 by the abnormally activated monocyte-macrophage system and excessive autoantibodies could also act synergistically with IL-17 produced mainly by Th17 to contribute the pathogenesis of RA (138). A randomized clinical trial has also implicated the strategy of treating RA patients by neutralizing IL-17 with monoclonal antibodies (139). Monocytes/Macrophages are also considered to mediate the inflammation of synovitis via other pathways and produce inflammatory cytokines including IL-6/12/15/18/23, reactive oxygen and nitrogen besides TNF-α and IL-1 (136). Bone diseases in RA patients often present as bone erosions mediated by the over-activated osteoclasts. There are also evidence pointing out that the conversion from monocytes/macrophages to osteoclasts is directed by macrophage-colony-stimulating factor (M-CSF) and receptor activator of nuclear factor κ-B ligand (RANKL) (140). In general, the overall inflammation in RA patients involves both adaptive and innate immune system, however the initial trigger remains unknown. Here we focus on the potential role of microbiota and innate immune system in the pathogenesis of RA.

In the history of the RA study, the establishment of the IL-1 receptor antagonist-deficient (*Il1rn*^−/−^) mouse model was the landmark of animal studies. The SPF *Il1rn*^−/−^ mice all developed RA-like autoimmune arthritis induced by T cells spontaneously within 5-month old. However, GF *Il1rn*^−/−^ mice, manifested no spontaneous arthritis symptoms, which strongly indicated that gut microbiota could somehow induce the inflammation reaction (141). Upcoming research on this mouse model revealed that the activation of specific TLRs by enteric bacteria is the key to initiate joint inflammation. An extra knockout of TLR2 in *Il1rn*^−/−^ mice could downregulate the expression level of FoxP3, a key transcription factor of Tregs. Afterwards, a reduction of the immune regulatory function of Tregs and an intensification of the inflammation reaction of arthritis occur. However, in the *Tlr4*^−/−^ *Il1rn*^−/−^ mouse model, the arthritis manifestation is weaker than that of the *Il1rn*^−/−^ mouse model. Researchers believed that TLR4 inhibited the proliferation and also the activity of Th17 so that the production of IL-17 was eventually downregulated (142). Hence, how the certain PAMP-TLR pairs and their specific microorganisms are related to the pathogenesis might be the next question to answer according to the previous studies.

Meanwhile, studies including ours have demonstrated that there are significant changes of fecal, salivary and dental microbiota diversity between RA patients and healthy population, which are also correlated with clinical disease markers and auto-antibody productions (134, 143–145). These findings suggest that gut microbiota might be crucial in the initiation of systemic inflammation reaction, especially in people with genetic susceptibility. And antigen mimicry to autoantigens including Sm and Fas from the lupus-enriched species was consolidated by *in vitro* experiments, which further suggest the possible role of GALTs and the local antigen presenting cells to present mimicking pathogenic antigens (134). There are also other studies showing that the over-reproduction of RA-prone species such as segmented filamentous bacteria (SFB) and lactobacillus sabotage the symbiosis status of commensal flora. And accumulation of SFB- and lactobacillus-related molecules including ATP, serum amyloid protein A, and CCL5 would further contribute to the induction of auto-reactive Th17 and Th1 cells. These auto-reactive helper T cells migrate body-wide and triggered an extensive downstream autoimmune response (146).

The inflammasome pathway was recently thought to be crucial in the pathogenesis as well (147). Inflammasomes are vitally important components of the innate immune system, which consists of a group of multi-protein complexes. Inflammasomes can recognize damage related molecular pathogens and further induce the production of pro-inflammatory cytokines like IL-1β and IL-18 (148), which plays pivotal roles in control of chronic inflammation. A study pointed out that the susceptible gene *A20* of RA regulates inflammasome negatively and the *A20* knockout mice could develop arthritis-like symptoms (149), which emphasized the significance of inflammasome on RA pathogenesis. After depletion of the another inflammasome related gene *NLRP6*, its pathway through ASC1 and caspase-1 is blocked and the IL-18 is consequently downregulated. Subsequently, an overgrowth of *prevotella* species occur, worsening the clinical manifestation of colitis (which is the disease model the group chose) (147). Intriguingly, the expansion of *prevotella* is also linked to RA susceptibility according to population studies including our unpublished data (150, 151). Moreover, the study found out that horizontal transfer of gut microbiota by housing wild-type and inflammasome-deficient mice together, the wild-type animals also developed similar phenotypes (147), indicating that the “crime specie” *prevotella* could potentially initiate gut inflammation even without genetic predisposition in mice. However, the reason why these mice developed no signs of arthritis is still unclear. The mechanism that *prevotella* contribute to the pathogenesis of RA also remained unclarified. On the other hand, population studies on RA patients showed that there are mutations of *NLRP3* and *CARD8* (that consists NLRP3 inflammasome complexes) which are also related to RA susceptibility, disease activity and response rate to TNF-antagonist treatments (152–155). Polymorphisms of inflammasome-related genes in RA patients might partially explain the accumulation of specific bacteria species in human, and additional research is required to explore the biology underlying.

In summary, the dysbiosis of gut microbiota could cause the alteration of the local innate immune system, such as the TLR pathway or inflammasome, which would lead the changes in systemic immune system and result in an autoreactive immune response. However, the certain species that contribute to the disease and how the dysbiosis occurred remains to be clarified. People are also questioning whether the dysbiosis is a result of systemic immune alteration or the primary changes in the pathogenesis.

### 3.2. Spondyloarthritis

Another systemic autoimmune disease that also affects joints is spondyloarthritis (SpA). SpA is considered to have a strong correlation with genetic predisposition and environmental exposure. Susceptible genes such as HLA-B27 and ERAP 1/2 have already been recognized to be closely related to the disease.

How gut microbiota in collaboration with local immune changes can contribute to the pathogenesis of SpA were completely a novel topic, and little is known in this area. However, according to some population studies carried out recently which found out more novel polymorphic genes that were possibly related to SpA, such as RUNX3 (156, 157) and TBX21 (158). These findings might enlighten us on the importance of ILCs in the pathogenesis of SpA. Runx3 is a pivotal transcription factor that induces RORγt and AHR, which guides the differentiation of ILC3 precursors to IL-17A and IL-22 expressing mature ILC3 (159). The transcription factor T-bet has already been found to be crucial in the development and differentiation ILC1. Mutation of the two genes might cause malfunctioning of the two populations. Moreover, T-bet was observed to be upregulated in SpA patients. Inhibition of T-bet could strongly inhibit the progression of disease in SKG mouse model (158).

On the other hand, a study analyzing the immune cell composition in the gut and synovial fluid of SpA patients revealed that accumulation of a subset of ILC3 which expresses NKp44 is an important source of IL-17A and IL-22, potentially contributing to the chronic inflammation (160, 161). Moreover, the study revealed that the gut NKp44+ ILC3 might re-enter the circulation pool and home to synovium via integrin α_4_β_7_. Hence, the gut inflammation in SpA which might be related to gut microbiota alterations could further seed inflammatory immune cells in synovium to add on the chronic inflammation.

Therefore, the T-bet related immune reactions involving ILC1, NK cells and certain population of T cells could be the next question to answer. As to Runx3, its mutation could affect the production of IL-17A and IL-22 by NKp44+ ILC3. How they are linked to changes of gut microbiota and disease progression could be essential to elucidate the pathogenesis of SpA.

### 3.3. Systemic Lupus Erythematosus

Systemic lupus erythematosus (SLE) is another systemic autoimmune disease that could affect almost every organ and tissue in the human body and could lead to severe organ failure or even death. Considerable efforts have been put into the research of SLE. People have understood that the eventual overproduction of self-reactive autoantibodies is the finale of pathogenesis. The hallmark of SLE is the failure of immune tolerance to self-antigens which are likely to be originated from microbial antigen mimicry. Aberrant gut immunity and dysbiosis could be closely related, both contributing to the loss of tolerance. In lupus patients or lupus-prone mouse models, studies have discovered various alteration of both immune cells and altered dynamics of gut microbiota (162). However still, little is conclusive about the whole picture of the pathogenesis of SLE.

In lupus patients, cNK cells in the periphery have less frequency and they presented reduced cytotoxicity as described above (89). Phagocytes like macrophages and dendritic cells produce increased level of cytokines including IL-6, IL10, and TNFα (163). Moreover, an animal study showed that female SNF1 mice display enhanced expression of PRRs like TLR-7 and TLR-8 on gut mucosa comparing to males, which suggests an explanation to the gender inequality in the incidence of lupus (164). Whether these immunological changes are consequences or cause of dysbiosis is still under discussion. Anyhow, dysbiosis at the preclinical stage in people with genetic predispositions is believed to contribute to the breakdown of self-tolerance (165).

A study utilizing the MRL/lpr and B6/lpr lupus-prone mouse model revealed that the SCFAs producing species including *Lachnospiraceae* and *Clostridiaceae* are enriched in the disease model comparing to controls (166). Additionally, dysbiosis in the gut is proven to interrupt the regulatory function of ILC3 and could exacerbate the response of self-reactive T cells, as mentioned in the previous chapter (109, 110). Moreover, the reduction ratio of Firmicutes/Bacteroidetes (F/B) in lupus patients is recently linked to the shift to Th17-axis immune response *in vitro* (167). And at the meantime, alterations of SCFAs and serum free fatty acids were also observed, which potentially might be linked to the F/B imbalance (168). The impact of SCFAs on human immune system is broad and somewhat controversial. As discussed in previous chapters, SCFAs as crucial microbiota signals, condition innate immune cells for their proper immune responses against pathogens and could contribute to local inflammation. However, SCFAs could also augment the production of anti-inflammatory cytokines by phagocytes via epigenetic regulation. In this case, how the alteration of SCFAs in lupus patients contributes to disease pathogenesis still needs thorough investigations.

Last year, the translocation of *Enterococcus gallinarum*, a specie of commensals, from gut to liver was found to induce Th17-axis over-reaction and worsen the lupus-like manifestations, in both the lupus-prone mice (F_1_ of NZW × BXSB) and GF C57BL/6 mice (Figure 1F). And strikingly, the specie was also positive in the liver biopsy of lupus patients (169). The findings suggest that breakdown of the mucosal integrity of gut, due to genetic predispositions such as TLR deficiency or the dysbiosis status, could contribute to the translocation of microbiota, whether pathogenic or symbiotic. The exposure of orthologs either *in situ*, in mLNs or in liver, could potentially induce the hazardous Th17-response in the pathogenesis of SLE.

In general, lupus, manifested by multi-system lesions mediated by aberrant production of autoantibodies, could be a spectrum of linked heterogenous conditions rather than one simple disease. Hence, studies conducted so far may have introduced unknown confounding factors. Meanwhile, the cohort size involved in studies might also limit the significance. However, abundant studies, especially animal works, into gut microbiota and lupus also shed light on that gut microbiota could be the missing link in the pathogenesis of SLE. Studies further to look at how the alteration of gut microbiota contributes to changes in the immune system are required in order to tackle SLE.

## 4. Concluding Remarks

Microenvironment in the gut is sophisticatedly shaped by the reciprocal interactions between gut microbiota and the local innate immune system. Commensal bacteria are required for structural development of GALTs and priming various immune cells for proper immune function. The mechanisms include signaling pathways through PRR-PAMP recognition, antigen exposure and presentation and epigenetic modulation via metabolic by-products such as SCFAs. The current understanding points out that commensal flora and competent immune system are crucial for the build-up of self-tolerance, whereas dysbiosis might alter local immune system and sabotage the tolerance. Several studies demonstrated that phagocytes such as macrophages and dendritic cells, as well as ILCs, are crucial in restraining self-reacting immune cells.

On the other hand, there are sufficient evidence that dysbiosis of gut microbiota occurs in various autoimmune diseases such as rheumatoid arthritis, spondyloarthritis, systemic lupus erythematosus. Change of gut microbiota could be a consequence of genetic predisposition and diet or a direct outcome of immune system disorders. However, there is also evidence proving that dysbiosis in the preclinical stage might initiate or exacerbate immune disorders.

The axis of “microbiota—innate immunity—disease” is not consolidated yet but might be a crucial pathogenesis mechanism in various diseases. Management at early stages to reverse the dysbiosis status by fecal transplantation or probiotics administration could possibly be a therapeutic strategy in combination with classical treatments to autoimmune diseases. However, only limited studies have been conducted and had contradictory conclusions. The feasibility and efficacy of such strategies remained unanswered and some ongoing large cohort clinical trials might provide more evidence in the near future. Taking one step further, deciphering the roles of gut microbiota and immune system could possibly prompt a huge leap in the treatment of autoimmune diseases.

## Author Contributions

YJ did the literature review and wrote the main body of the article. NH and XZ co-supervised on finishing this review. NH is an experienced immunologist in NK cell biology and supervised the corresponding chapters. XZ is an experienced clinical immunologist and rheumatologist and supervised the corresponding chapters. LW provided suggestions on chapters of dendritic cells and macrophages. All authors read and approved the final manuscript for publication.

### Conflict of Interest

The authors declare that the research was conducted in the absence of any commercial or financial relationships that could be construed as a potential conflict of interest.

## Abbreviations

DC: dendritic cells
IL: interleukin
IFN: interferon
cNK cells: conventional natural killer cells
SCFAs: short-chain fatty acids
TNF: tumor necrosis factor
IEC: intestinal epithelial cell
TSLP: thymic stromal lymphopoietin
MHC II: major histocompatibility complex class II.
